# Quantitative assessment of harmonic power doppler myocardial perfusion imaging with intravenous levovist™ in patients with myocardial infarction: comparison with myocardial viability evaluated by coronary flow reserve and coronary flow pattern of infarct-related artery

**DOI:** 10.1186/1476-7120-3-22

**Published:** 2005-08-18

**Authors:** Tomoko Tani, Kazuaki Tanabe, Minako Tani, Fumie Ono, Minako Katayama, Koichi Tamita, Shuichiro Kaji, Atsushi Yamamuro, Kunihiko Nagai, Kenichi Shiratori, Shigefumi Morioka, Yasuki Kihara

**Affiliations:** 1 Division of Cardiology, Kobe General Hospital, 4–6 Minatojima-Nakamachi, Chuo-ku, Kobe, Japan

## Abstract

**Background:**

Myocardial contrast echocardiography and coronary flow velocity pattern with a rapid diastolic deceleration time after percutaneous coronary intervention has been reported to be useful in assessing microvascular damage in patients with acute myocardial infarction.

**Aim:**

To evaluate myocardial contrast echocardiography with harmonic power Doppler imaging, coronary flow velocity reserve and coronary artery flow pattern in predicting functional recovery by using transthoracic echocardiography.

**Methods:**

Thirty patients with anterior acute myocardial infarction underwent myocardial contrast echocardiography at rest and during hyperemia and were quantitatively analyzed by the peak color pixel intensity ratio of the risk area to the control area (PIR). Coronary flow pattern was measured using transthoracic echocardiography in the distal portion of left anterior descending artery within 24 hours after recanalization and we assessed deceleration time of diastolic flow velocity. Coronary flow velocity reserve was calculated two weeks after acute myocardial infarction. Left ventricular end-diastolic volumes and ejection fraction by angiography were computed.

**Results:**

Pts were divided into 2 groups according to the deceleration time of coronary artery flow pattern (Group A; 20 pts with deceleration time ≧ 600 msec, Group B; 10 pts with deceleration time < 600 msec). In acute phase, there were no significant differences in left ventricular end-diastolic volume and ejection fraction (Left ventricular end-diastolic volume 112 ± 33 vs. 146 ± 38 ml, ejection fraction 50 ± 7 vs. 45 ± 9 %; group A vs. B). However, left ventricular end-diastolic volume in Group B was significantly larger than that in Group A (192 ± 39 vs. 114 ± 30 ml, p < 0.01), and ejection fraction in Group B was significantly lower than that in Group A (39 ± 9 vs. 52 ± 7%, p < 0.01) at 6 months. PIR and coronary flow velocity reserve of Group A were higher than Group B (PIR, at rest: 0.668 ± 0.178 vs. 0.248 ± 0.015, p < 0.0001: during hyperemia 0.725 ± 0.194 vs. 0.295 ± 0.107, p < 0.0001; coronary flow velocity reserve, 2.60 ± 0.80 vs. 1.31 ± 0.29, p = 0.0002, respectively).

**Conclusion:**

The preserved microvasculature detecting by myocardial contrast echocardiography and coronary flow velocity reserve is related to functional recovery after acute myocardial infarction.

## Introduction

In patients with myocardial infarction (MI), the distinction between irreversible fibrotic scar and akinetic but viable myocardium has important clinical implications. Several imaging techniques have been used to detect myocardial viability. Coronary blood flow reserve (CFR) has been established as a useful method for assessing microvascular function [1-3]. Previous studies revealed that the measurement of CFR in the infarct-related coronary artery might help to assess myocardial viability [4-6].

Kawamoto T et al. showed that coronary blood flow spectrum immediately after primary percutaneous transluminal coronary angioplasty (PTCA) by use of a Doppler guidewire reflected a greater degree of microvascular damage in the risk area, and was useful in predicting recovery of regional left ventricular (LV) function [7].

The diastolic deceleration slope of coronary flow velocity is steeper in patients with substantial 'no reflow' phenomenon assessed by myocardial contrast echocardiography (MCE). Recently, Shintani et al revealed that patients with a shorter deceleration half time (DHT) of diastolic coronary flow velocity have a poorer functional outcome among patients with anterior AMI and that the transthoracic Doppler echocardiography (TTDE)-determined DHT is a useful predictor of myocardial viability after anterior AMI [8].

The assessment of MCE with intracoronary or venous injection of ultrasound contrast agents composed of microbubbles has been shown to provide information on perfusion territories, collateral flow, infarct size, myocardial viability, and success of reperfusion therapy [9-12]. Recent advances in both of the contrast agents and ultrasound technology have enabled the improvement of detection of myocardial perfusion using intravenous contrast application. The evaluation of myocardial perfusion is an important component of the risk stratification of patients with ischemic heart diseases. Harmonic power Doppler imaging (HPDI) has emerged as a promising tool to detect myocardial perfusion after intravenous injection of contrast agents. MCE may be more versatile than perfusion scintigraphy for identifying the presence and extent of perfusion defects after MI. Recent studies indicated that HPDI can reliably detect myocardial perfusion at rest and during pharmacological stress [13,14]. Previous works, however, used qualitative analysis of the HPDI. Digital acquisition of HPDI should lend itself to quantitative analysis, which may be more accurate in distinguishing normal perfusion from mild defects. In addition, quantitative analysis offers the potential to measure flow reserve ratio noninvasively.

The aim of this study was to compare the MCE results with myocardial viability evaluated by CFR two weeks after AMI and CF of LAD at acute phase measured with high-frequency TTDE echocardiography and evaluated MCE with HPDI, CFR and CF in predicting functional recovery and LV remodeling.

## Methods

### Patient Population

This study included 30 patients (age 62 ± 10 years, 5 women and 25 men) with the first anterior AMI. All patients underwent successful revascularization by PTCA and stent placement in the acute phase (within 6 hours after the onset of chest pain). Inclusion criteria were typical anginal chest pain lasting > 30 min and ST-segment elevation of > 0.2 mV in at least two contiguous electrocardiographic leads. And inclusion criteria were also echocardiographic images of sufficient quality to allow adequate visualization of all myocardial segments from the apical views and angiographically documented patency of the infarct-related artery (TIMI grade 3 flow) on coronary angiograms taken in the repeated study. Six patients were excluded from analysis because of inadequate image quality of echocardiograpic images and coronary flow by TTDE.

Exclusion criteria were pregnancy, lactation, unstable angina and second or third degree atrioventricular block, bronchial asthma and systolic blood pressure < 90 mmHg. All patients gave written informed consent.

### Myocardial Contrast Echocardiography

Transthoracic 2-dimensional (2D) echocardiographic images were obtained with an ultrasound imaging system (Sonos 5500, Philips Medical systems, Andover, MA, USA). MCE was performed two weeks after AMI. The contrast agent used for this study was Levovist™, which is composed of galactose (mean diameter 1.2 μm). MCE was performed by administration of Levovist at a dose of 300 mg/dl by injecting 3 ml/3 sec using by Medrad Pulser™ (Ultrasound injection system, MEDRAD Service Department, Indianola, PA), followed by a 10 ml saline flushed. Contrast enhanced images using HPDI mode (scanhead transmit to receive frequency = 1.8:3.6 MHz) were acquired on intermittent mode at each pulse intervals of 4 cardiac cycles with the ultrasound transmission gated to the T wave of the electrocardiogram (i.e. end-systole). The dynamic range of this system is 40 dB. The mechanical index was set as high as possible to increase microbubble destruction. Ultrasound system gains were optimized at the beginning of the study and held constant for subsequent image acquisitions. The filter threshold was optimized to reduce the appearance of any color over the myocardium before contrast injection. The apical 4-chamber view was used to assess myocardial perfusion in all patients. We set the focus point at the mid-portion of the LV and kept constant for the quantitative analysis at baseline and during ATP stress. Images were stored on a 2.3 Gbytes MO disk.

We examined MCE under conditions at initial baseline and during an infusion of ATP. The dosage of ATP was selected of 0.15 mg/kg/min. Levovist was pushed into the intravenous infusion at baseline imaging and during ATP infusion for stress imaging. Since peak hyperemia begins 2 minutes after the ATP infusion, image acquisition was initiated 2 minutes after the start of the infusion. Since only 2–2.5 minutes of maximal hyperemia was available for imaging, HPDI was performed only in the apical views by obtaining 3 to 5 beats gated to every fourth cardiac cycle.

### Image Analysis of Echocardiogram

**Figure 1:**
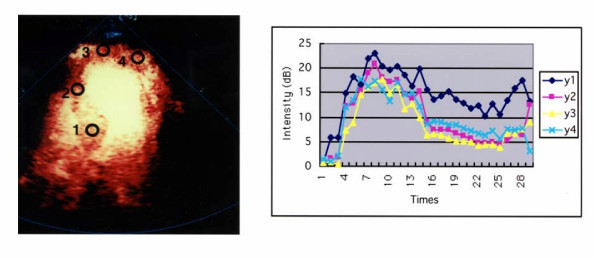
Quantitative analysis of harmonic power Doppler imaging. A. Four different regions of interest are drawn on the digital clip. The ROI can be individually repositioned in each frame to match the anatomy. B. The graph shows time-intensity curves of the mean Doppler information (dB) from the ROIs. y1 = basal septum, y2 = mid septum, y3 = apex, y4 = apical portion of lateral segment.

The analysis was performed off-line using QuantiCon software (Echo Tech 3D Imaging Systems, Germany) for the quantification of contrast based ultrasound images. Regions-of-interest (ROIs) were placed in the basal septum, mid septum, apex and apical portion of the lateral segment. The ROIs can be individually repositioned in each frame to match the anatomy of the unaligned images. The information is displayed as the number of scatters that have moved in the power mode. Thus, intensity curves of the mean Doppler information in the ROIs can be plotted overtime using this software. Peak intensities of color pixels (dB) were then measured in 4 different segments of the LV. The peak intensity ratios of the risk area (apical segment) to the control area (PIR) at rest and during hyperemia were calculated. The control region was set in the basal septum in this study (Fig. 1).

### Doppler Echocardiographic Studies

We performed Doppler measurements of LAD flow velocity by TTDE within 24 hours after successful recanalization. Doppler echocardiographic examinations were performed with an SONOS 5500 digital ultrasound system with a broadband high frequency (5-12MHz) transducer (S12) and a Logic 500 digital ultrasound system with a frequency of 8 MHz (GE medical system). The color gain was adjusted to provide optimal images. The acoustic window was around the midclavicular line in the fourth and fifth intercostal. The ultrasound beam was transmitted toward the heart to visualize coronary blood flow in the LAD by color Doppler echocardiography. With a sample volume positioned on the color signal in the LAD, Doppler spectral tracings of flow velocity in the LAD were recorded by fast Fourier transformation analysis. All studies were continuously recorded on 1/2-inch super-VHS videotape for off-line analysis.

**Figure 2:**
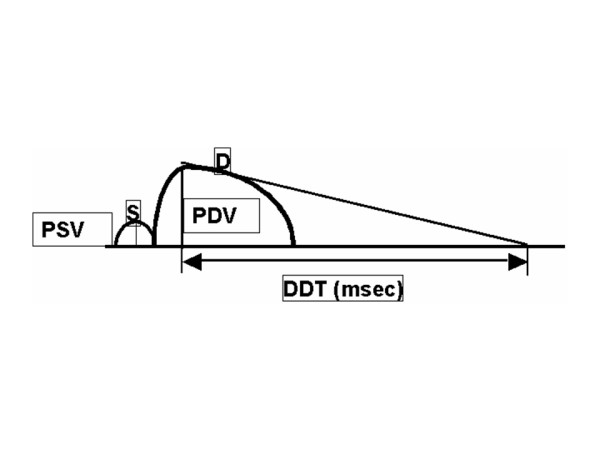
Measurement of parameters from systolic and diastolic flow velocity patterns. By tracing the contour the coronary flow velocity wave form, we measured the peak diastolic velocity (PDV: cm/sec), mean diastolic velocity (MDV: cm/sec), peak systolic velocity (PSV: cm/sec) and deceleration time of diastolic flow velocity (DDT: msec).

The digitized coronary blood flow velocity spectrum provided the following parameters: peak systolic velocity (PSV: cm/sec), mean diastolic velocity (MDV: cm/sec), peak diastolic velocity (PDV: cm/sec), and deceleration time of diastolic flow velocity (DDT: msec). Retrograde flow was calculated as a negative value (Fig. 2).

### CFR Measurements by TTDE

We measured CFR of LAD two weeks after AMI at the same time of MCE. At first, we recorded baseline spectral Doppler signals in the distal portion of the LAD. Then, ATP was administered for two minutes and spectral Doppler signals were recorded during hyperemic conditions. All patients had continuous heart rate and ECG monitoring. Blood pressure was recorded at baseline, every minute during ATP infusion and at recovery. One experienced investigator who was unaware of the other patient data analyzed each study. Measurements were performed off-line by tracing the contour of the spectral Doppler signal using the computer incorporated in the ultrasound system. Mean diastolic velocity (MDV) and peak diastolic velocity (PDV) were measured at baseline and peak hyperemic conditions. CFR was defined as the ratio of hyperemic to basal peak diastolic coronary flow velocity.

### Two-dimensional Echocardiographic Measurements of LV Function at Baseline

Before CFR measurements by TTDE, we measured LV wall (septal and posterior wall thickness) at end diastole by two-dimensional echocardiography. LV volume measurements were performed according to the recommendation of the American Society of Echocardiography. Apical two- and four-chamber views were obtained at baseline. End-diastolic and end-systolic LV volumes were computed by use of modified Simpson's method (method of disks). Furthermore, we assessed regional wall motion at rest on the basis of 16 segments of the LV as recommended by the American Society of Echocardiography.

### Coronary Angiography

Left heart catheterization was performed by the femoral approach after local anesthesia induced with 0.5% lidocaine. Biplane left ventriculography was performed after injection of 4000 IU IV heparin to assess LV wall motion and to measure LV volume by the area-length method in the acute phase and 6 months after AMI. Selective coronary angiography was carried out by the Judkins technique after an intravenous injection of 3 mg of isosorbide dinitrate. Coronary angiography was analyzed quantitatively with the use of videodensitometric analysis performed with a commercially available system (CAMAC-300, Goodman, Inc.) in the manner previously reported [15].

### Statistical Analysis

Data were expressed as a mean ± standard deviation. Continuous data between groups were compared by student *t*-test, correlation between Doppler parameters and PIR was performed using linear regression analysis. Statistical significance was defined as p < 0.05.

Receiver operating characteristics (ROC) curve analysis was performed. Analyses were done with SPSS for Windows software.

## Results

### Patient Characteristics

**Figure 3:**
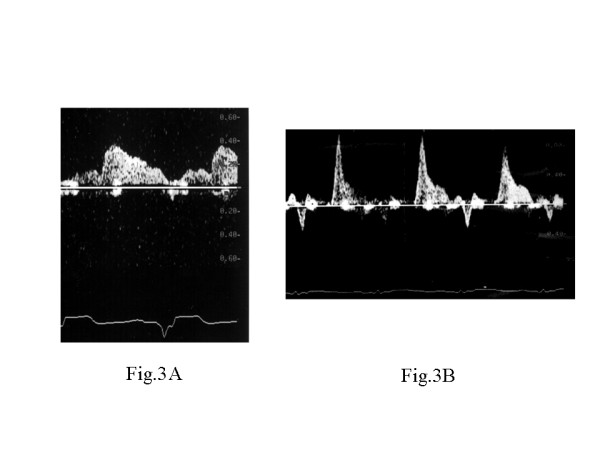
Representative coronary flow pattern in Group A (**A**) and Group B (**B**).

The clinical characteristics of the patients are summarized in Table 1. The mean time interval between the MCE studies and coronary angiography was 16 ± 2 days. There were no clinical events among these examinations in any of the patients. In accordance with previous findings[7], patients were divided into two groups based on CF pattern: group A (n = 20) with DDT ≧ 600 msec and group B (n = 10) with DDT ≧ 600 msec. Representative coronary flow pattern is shown in Fig. 3. All patients received conventional drug therapy based on individual needs, as determined by the attending physician.

**Table 1:** Patient Characteristics

	Group A	Group B
Number	20	10
Age	63 ± 12	64 ± 10
Men (%)	17 (85%)	8 (80%)
Cardiovascular risk factors		
Diabetes Mellitus	7	4
Hypertension	11	6
Smoking	12	7
Total Cholesterol (mg/dl)	200 ± 38	189 ± 13
HDL-cholesterol (mg/dl)	43 ± 8	48 ± 12
Triglyceride (mg/dl)	155 ± 94	105 ± 44
Visible Collaterals to Infarct-related artery	5/20	3/10
Referece Diameter (mm)	3.4 ± 0.7	3.1 ± 0.5
TIMI 3 flow after PCI	18/20	5/10*

*p < 0.01

PCI: Percutaneous Coronary Interventional Therapy

TIMI: Thrombolysis in Myocardial Infarction

### Myocardial Contrast Echocardiography

**Figure 4:**
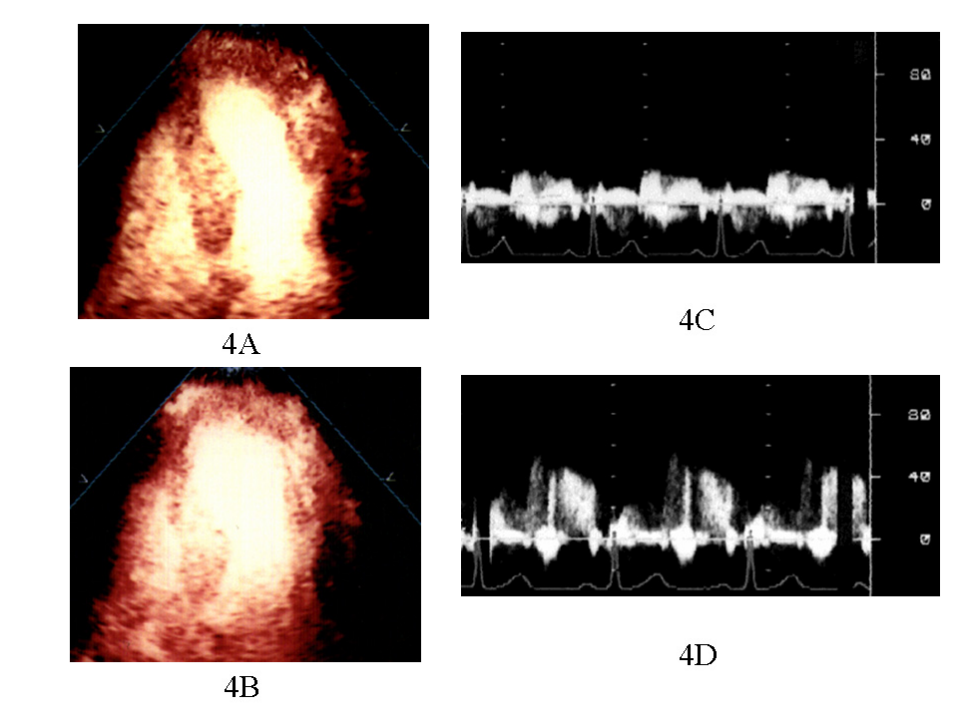
The results of MCE and CFR in a patient with Group A. 1) HPDI showing increase in contrast signals in the anteroseptal and apical lesion in 4-chamber view during ATP stress image (**B**) when compared with baseline image (**A**). 2) PDV in the infarct-related artery increased during ATP stress (**D**) compared with baseline (**C**) by TTDE.

**Figure 5:**
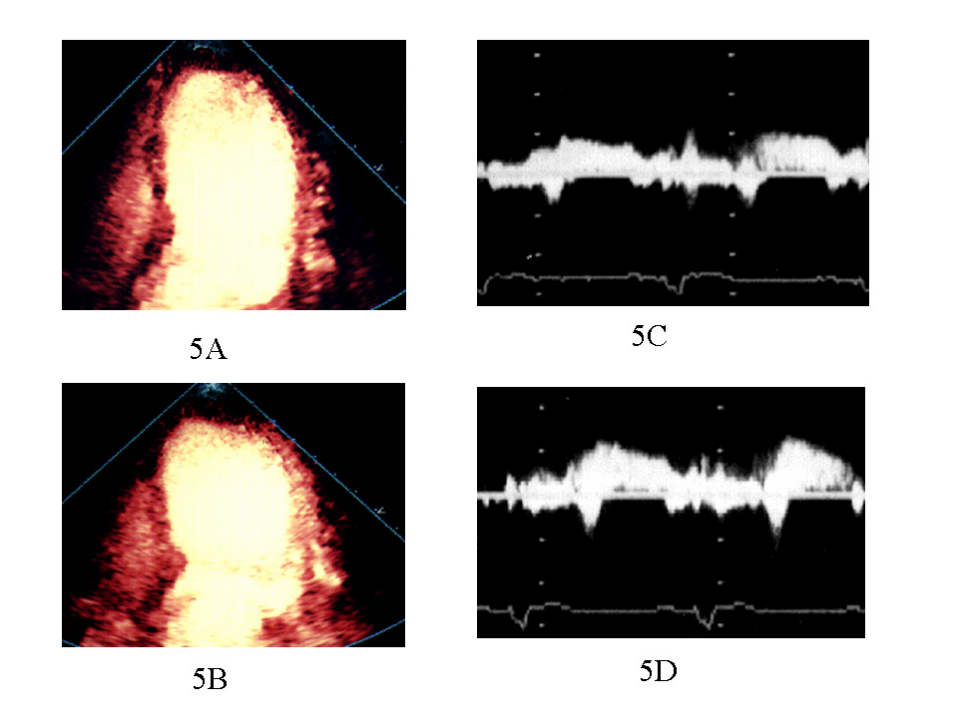
The results of MCE and CFR in a patient with Group B. 1) HPDI showing resting apical perfusion defect (**A**) and without improvement of perfusion during ATP stress (**B**) in the anteroseptal and apical lesion. 2) PDV in the infarct-related artery slightly increased during ATP stress (**D**) compared with baseline (**C**) by TTDE

There were no significant changes in the average heart rate (65 ± 4 vs. 77 ± 9, p = 0.08), systolic blood pressure (118 ± 11 vs. 94 ± 26, p = 0.21), and diastolic blood pressure (59 ± 8 vs. 58 ± 15, p = 0.96) after intravenous ATP administration in all patients. ATP did not cause significant side effects in the study patients. Figure 4 shows a representative perfusion image in a patient with anterior AMI of Group A. MCE revealed mild perfusion defect in the risk area and improved perfusion defect during hyperemia. Figure 5 shows a representative perfusion image in a patient with anterior AMI of Group B. There was a contrast perfusion defect in the risk area by MCE. In patients of Group A, PIR both at rest and during hyperemia of HPDI were significantly higher compared with those in patients of Group B (at rest: 0.668 ± 0.178 vs. 0.248 ± 0.015, p < 0.0001: during hyperemia 0.725 ± 0.194 vs. 0.295 ± 0.107, p < 0.0001).

According to receiver operating characteristics curve analysis, the optimal cutoff value to identify myocardial viability by MCE was 861 msec for DDT (sensitivity 87%, specificity 80%) and 1.8 for CFR (sensitivity 87%, specificity 73%).

### Coronary Blood Flow Pattern and Coronary Flow Reserve

FR in patients of Group A was significantly higher compared with CFR of Group B (2.60 ± 0.80 vs. 1.31 ± 0.29, p < 0.0001). In patients without a perfusion defect in the risk area by MCE, CFR was > 2.0 (Table 2). Representative imagings of MCE and CFR in two groups were shown in Fig. 4 and Fig. 5.

**Table 2:** Differences of Parameters between Two Groups

	Group A	Group B
PIR at rest	0.668 ± 0.178	0.248 ± 0.015^#^
PIR at ATP stress	0.725 ± 0.194	0.295 ± 0.107^#^
EDV in the acute phase (ml)	112 ± 33	146 ± 38
EDV at follow-up (ml)	114 ± 30	192 ± 39*
EF in the acute phase (%)	50 ± 7	45 ± 9
EF at follow-up (%)	52 ± 7	39 ± 9*
LVWMI in the acute phase	2.1 ± 0.7	2.4 ± 0.3
LVWMI at follow-up	1.7 ± 0.4	2.4 ± 0.3*
Peak creatine kinase (U/L)	2734 ± 868	6198 ± 2265*
CFR	2.60 ± 0.8	1.31 ± 0.29*

^#^p < 0.05, *p < 0.01

PIR, peak intensity ratio; EDV, end-diastolic volume; EF, ejection fraction; CFR, coronary blood flow reserve; LVWMI, left ventricular wall motion index

DDT of Group A was significantly longer than that of Group B (985 ± 136 vs. 222 ± 115, p < 0.0001). CFR was correlated with DDT (p < 0.0001, r^2^Q = 0.566).

PSV of Group A was significantly larger than that of Group B (12.2 ± 2.81 vs. -22.0 ± 23.5, p = 0.0006). PDV of group A was significantly smaller than that of Group B (23.5 ± 6.19 vs. 45.1 ± 21.8, p = 0.012).

According to present/absent post-infarct LV remodelling at follow-up, I divided two groups (Table 3).

**Table 3:** Differences of Parameters between Two Groups

	Group 1	Group 2	p
PIR at rest	0.700 ± 0.161	0.334 ± 0.162	0.0001
PIR at ATP stress	0.736 ± 0.151	0.358 ± 0.057	0.0007
Peak creatine kinase (U/L)	1526 ± 1021	3724 ± 684	0.02
DDT	997 ± 161	427 ± 350	0.0003
CFR	2.35 ± 0.50	1.71 ± 0.72	0.03

Group 1: LV remodeling(-)

Group 2: LV remodeling(+)

### Left Ventricular Volume and Function

In the acute phase, there were no significant differences of LVEDV and EF between the two groups.

LVEDV of Group B was, however, significantly larger than that of Group A, and EF of Group B was significantly decreased as compared with EF of Group A 6 months after AMI (Table 2).

### LV Wall Motion Recovery and CFR, CF Pattern

**Figure 6:**
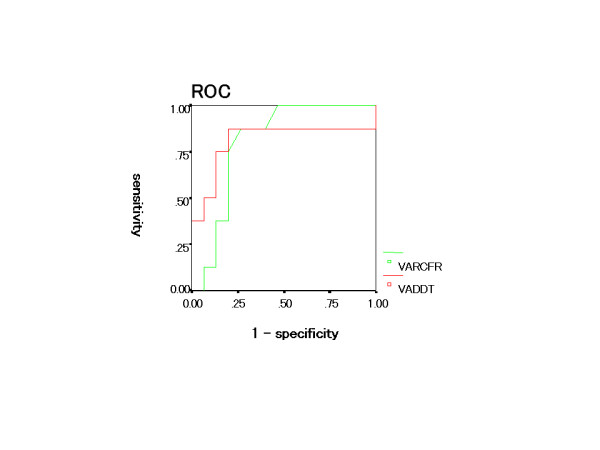
The result of receiver operating characteristics curve about DDT and CFR to predict wall motion recovery.

According to receiver operating characteristics curve analysis, the optimal cutoff value to predict wall motion recovery was 809 ms for DDT (sensitivity 87%, specificity 73%) and 2.0 for CFR (sensitivity 87%, specificity 87%) (Fig. 6).

## Discussion

In patients with reperfused anterior AMI, a shorter DDT of the LAD flow detected within 24 hrs after coronary reperfusion and CFR less than 1.75 were feasible predictors of poor functional outcome. These parameters correlated with the quantitative results by HPDI with intravenous MCE. TTDE provides a simple and promising means of noninvasive assessment of myocardial viability in patients with reperfused anterior AMI.

### Coronary Flow Pattern

Recent studies with intracoronary MCE have shown that about one fourth to one third of patients with AMI treated with primary PTCA have an inadequate tissue perfusion (no-reflow phenomenon) despite angiographically successful coronary recanalization [16]. Characteristic coronary flow velocity pattern (systolic retrograde flow and rapid deceleration of diastolic flow) by using a Doppler guidewire is associated with the "no-reflow" phenomenon [17]. This "no-reflow" phenomenon is thought to be the result of microvascular dysfunction.

In patients with MCE no reflow, the coronary microvasculature was profoundly damaged and it seemed that microvascular impedance increased and the intramyocardial blood pool decreased. The coronary microvasculature would be diffusely obstructed in patients with MCE no reflow. Early systolic retrograde flow (ESRF) has been reported to be observed frequently in patients with no reflow in case of AMI after successful recanalization and was explained by an occluded coronary microvasculature.

Kawamoto et al. showed that the degree of reduced systolic antegrade flow or the deceleration time of diastolic flow reflected the degree of microvasculature damage and was predictive of residual myocardial viability [7]. They revealed that low average peak velocity and rapid DDT of coronary blood flow spectrum immediately after primary PTCA reflected a greater degree of microvascular damage in the risk area and analysis of coronary blood flow spectrum immediately after primary PTCA by use of a Doppler guidewire was useful in predicting recovery of regional LV function.

Recently, Wakatsuki et al. revealed that the coronary flow velocity pattern measured immediately after successful primary stenting is predictive of the recovery of regional and global LV function in patients with AMI [18]. The changes of regional wall motion score (RWM) and EF were significantly greater in the non-ESRF group than it was in the ESRF group. These authors reported that ESRF is a parameter predicting poor functional recovery of LV wall motion. They explained that decreased extravascular pressure of the infarcted myocardium during systole might increase the apparent systolic flow, and increased vascular resistance might decrease the diastolic antegrade flow in mildly to moderately damaged myocardium.

In our study, there were 6 patients with early systolic retrograde flow. They had no myocardial perfusion in infarcted area by HPDI and revealed poor CFR. LV wall motion in all these patients did not recover at follow-up. The obstruction of the microvasculature with subsequent high impedance results in the inability to squeeze blood forward into the venous circulation during systole. It is consequently pushed back to the epicardial coronary artery to produce early systolic retrograde flow. The reduced intramyocardial blood pool, which fills rapidly during diastole, has been used to explain the rapid decline of diastolic velocity.

Recently, Lepper et al. reported that the coronary flow reserve immediately and 24 hr after PTCA for AMI relates to myocardial perfusion determined by MCE and LV function in four weeks [19]. From our results, the patients with significantly altered coronary flow pattern, which showed rapid DDT, were found to have subsequent depression of LV function at follow up, confirming the prognostic importance of altered coronary blood flow patterns reported in previous studies [6,7]. This study confirms recent reports on the difference in coronary blood flow pattern between patients with and without reperfusion determined by MCE.

There were no reports that were compared coronary blood flow velocity pattern and CFR by TTDE with intravenous MCE by using HPDI. Our study revealed that DDT at acute phase correlated with microvascular integrity by HPDI and with CFR after two weeks. The quantitative analysis by HPDI revealed the degree of microvascular dysfunction. HPDI is a feasible technique for the detection of a myocardial perfusion defect in patients with coronary artery disease after a venous injection of contrast agent. Previous works, however, used qualitative analysis of the HPDI [14]. In the present study, we performed quantitative analysis of HPDI at rest and during hyperemia in patients with AMI.

We used Levovist, which is sensitive to microbubble destruction at diagnostic ultrasound frequencies [19]. Several recent studies have demonstrated that the feasibility of applying this contrast agent to the assessment of myocardial perfusion by using HPDI [21].

In patients who had myocardial viability, the peak intensity of the risk area was 0.668 ± 0.178 dB at rest. In patients who had no myocardial viability in the risk area, the curves of intensity showed no increase and peak intensity was 0.248 ± 0.015 dB at rest. During ATP induced hyperemia, peak intensities were increased in the segments with preserved myocardial integrity. In the segments without preserved myocardial integrity, however, there was no change in peak intensities. We diagnosed quantitatively myocardial viability using by HPDI. HPDI is strictly dependent on microvascular integrity and PIR shows quantitatively the degree of microvascular damage. There were significant differences of LV function at follow-up between two groups. Our data may provide additional information from the point of coronary flow pattern.

### Coronary Artery Flow Reserve

Our main findings were that the myocardial perfusion status assessed by HPDI at rest and during ATP stress corresponds closely to CFR and DDT of CF. CF at acute phase and CFR two weeks after AMI onset correspond to left ventricular remodelling at chronic phase.

A previous study showed that, in patients with myocardial infarction, CFR is inversely correlated with the extent of myocardial infarction and directly correlated with the improvement in wall motion contractility during the recovery period [4,6]. A value above 1.75 is associated with an improved wall motion index. Reduction of MCE perfusion defects were associated with improvement of CFR, whereas persistent MCE perfusion defects were associated with unchanged depression of CFR, indicating a relation between microvascular integrity assessed by CFR and by intravenous MCE [22].

There were no reports that had been investigated the relationship between CFR and DDT of LAD. From our study, CFR correlated with DDT. CFR and DDT revealed the degree of microvascular damage in myocardium.

The patients with MCE no reflow, which had no viability in the infarcted area by HPDI, showed poor functional outcomes and left ventricular remodeling. The detection of viability with HPDI and CFR were useful for predicting of LV remodeling.

### Study Limitations

We compared the myocardial opacification obtained by HPDI with CFR and DDT of LAD in patients after PTCA for AMI. The machine settings used in this study relate to the best knowledge at the time of study initiation. Optimal echocardiographic machine settings for MCE are rapidly evolving and are dependent on the applied contrast agent. Thus, it is very challenging to set up and adhere to a study protocol in a field in which knowledge of how to use an evolving technology is improving very quickly.

The differences in the shell structure as well as gas compositions of different microbubbles are likely to influence their efficacy for myocardial perfusion assessment by HPDI. Unfortunately, real-time perfusion imaging is not available on the instrument used in this study. Using real-time assessment of perfusion defects, we can diagnose both wall motion and myocardial perfusion at the same time [23].

We could not perform MCE study immediately after the primary angioplasty. This analysis included only a limited number of patients.

Coronary flow reserve is related to the microcirculatory status, which will be either indirectly affected by epicardial coronary stenosis or directly affected by a previous myocardial infarction and other related factors.

The coronary flow pattern may be influenced by left ventricular pressure. But we didn't compare CFR and coronary flow pattern with the left ventricular pressure. Further study is needed to clarify this issue. Systolic reversal flow is a specific indication of the no reflow phenomenon, but it was sometimes difficult to record the complete Doppler spectral envelope throughout the entire cardiac cycle by TTDE because of the systolic heart motion.

## Conclusion

Coronary flow velocity patterns relate to myocardial perfusion determined by intravenous MCE and CFR by TTDE.

Intravenous MCE and intermittent harmonic power Doppler imaging has the potential to noninvasively identify myocardial viability in patients with a previous anterior MI. Quantitative assessment of microvascular integrity corresponds to the evaluation of the microcirculation by CFR using transthoracic echocardiography.

The assessment of microvascular damage by HPDI, CFR and CF corresponds to left ventricular remodeling.

DDT and PIR were more useful in detecting functional recovery.

## List of Abbreviations

AMI-acute myocardial infarction

MCE-myocardial contrast echocardiography

CFR-coronary blood flow reserve

CF-coronary artery flow pattern

LV-left ventricular

TTDE-transthoracic Doppler echocardiography

HPDI-harmonic power Doppler imaging

PIR-peak intensity ratios of the risk area to the control area

LAD-left anterior descending artery

DDT-deceleration time of diastolic flow velocity

## Competing interests

The author(s) declare that they have no competing interests.
